# Crippling Violence: Conflict and Incident Polio in Afghanistan

**DOI:** 10.1371/journal.pone.0149074

**Published:** 2016-03-09

**Authors:** Alison Norris, Kevin Hachey, Andrew Curtis, Margaret Bourdeaux

**Affiliations:** 1 Division of Epidemiology, College of Public Health, Ohio State University, Columbus, Ohio, United States of America; 2 College of Medicine, Ohio State University, Columbus, Ohio, United States of America; 3 GIS, Health & Hazards Lab, Department of Geography, Kent State University, Kent, Ohio, United States of America; 4 Brigham and Women’s Hospital, Division of Global Health, Boston, Massachusetts, United States of America; Nottingham University, UNITED KINGDOM

## Abstract

**Background:**

Designing effective public health campaigns in areas of armed conflict requires a nuanced understanding of how violence impacts the epidemiology of the disease in question.

**Methods:**

We examine the geographical relationship between violence (represented by the location of detonated Improvised Explosive Devices) and polio incidence by generating maps of IEDs and polio incidence during 2010, and by comparing the mean number of IED detonations in polio high-risk districts with non polio high-risk districts during 2004–2009.

**Results:**

We demonstrate a geographic relationship between IED violence and incident polio. Districts that have high-risk for polio have highly statistically significantly greater mean numbers of IEDs than non polio high-risk districts (p-values 0.0010–0.0404).

**Conclusions:**

The geographic relationship between armed conflict and polio incidence provides valuable insights as to how to plan a vaccination campaign in violent contexts, and allows us to anticipate incident polio in the regions of armed conflict. Such information permits vaccination planners to engage interested armed combatants to co-develop strategies to mitigate the effects of violence on polio.

## Background

Poliomyelitis, a viral disease spread via fecal contamination of food or water sources, has been a source of death and disability worldwide for millennia. Acute paralytic disease, a condition seen in 1% of poliomyelitis infections, strikes primarily in early childhood, and can cause devastating life-long disability. In 1980, global paralytic polio incidence was approximately 400,000 cases annually [1]. In 1988, recognizing the massive global disease burden, the World Health Organization (WHO) outlined a plan for global eradication by the year 2000 [2]. The subsequent mass immunization campaigns enjoyed tremendous success: the disease was eradicated from the Americas by 1994, Europe followed in 2002, [3] and fewer than 3,000 cases were reported worldwide in 2000 [4].

While these numbers indicate that eradication of polio is possible, armed conflict has stymied progress in several key regions. In 2006, four countries had endemic polio: Nigeria, India, Pakistan, and Afghanistan [5]. Coordinated efforts to improve relations with marginalized ethnic and religious groups led to an increase in vaccine penetration, particularly in Nigeria and India [6]. Nigeria reported a 90% case reduction in 2010 alone [7], while January 13, 2015, marks four years without any paralytic disease in India [8].

**Fig 1:**
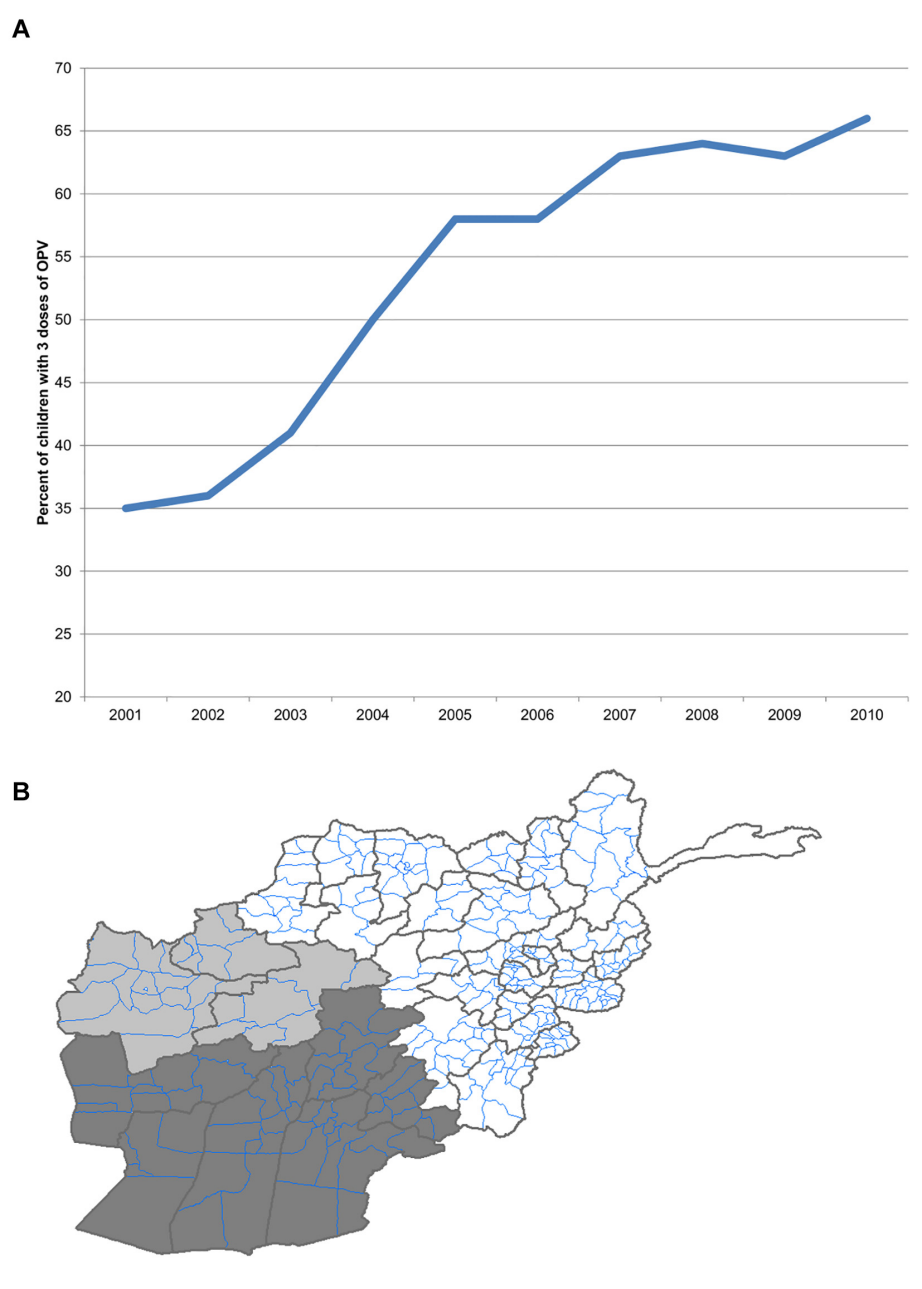
Vaccine coverage in Afghanistan. (A) Afghanistan national rate of oral polio vaccine coverage (2001–2011). Coverage was assessed via parental recall of children with non-polio acute flaccid paralysis. (B) Vaccine coverage in Afghanistan as of November 2011, by region. Dark grey represents 26% in the South Region (plus Farah province), light grey represents 71% in the West Region (minus Farah province), white represents 80% in the rest of the country.

The reverse is true in Afghanistan. Since the US-led invasion in 2001, there has been a dramatic increase in the number of cases of paralytic poliomyelitis from 11 cases in 2001, to 80 in 2011. [9]. However, WHO-UNICEF has estimated that national vaccination coverage rates have climbed from 35% in 2001 to 66% in 2011 (Fig 1A) [10]. Although national average coverage rates have increased, [10] regional disparities are substantial. In the Farah province and South region (Nimruz, Helmand, Kandahar, Zabul and Oruzgan provinces), only 26% of children aged 6–23 months had received the full 3 doses of oral polio vaccine (OPV) in 2010. This 26% coverage rate was an average for the region; it is reasonable to believe that areas with conflict would have had lower rates. The Western region (excluding Farah province) has achieved 71% coverage, and the rest of the country has reached 80% (Fig 1B) [11].

Designing effective public health campaigns in areas of armed conflict requires a nuanced understanding of how violence impacts the epidemiology of the disease in question. In the past, mathematical models of polio spread have been used to predict polio outbreaks, to plan subnational immunization days, and to ensure adequate vaccine stocks [12]. While many recent publications regarding polio eradication acknowledge the difficulties that the on-going conflict in Afghanistan cause for polio programs [5], [13], [14], there are few quantitative examinations of the relationship between violence and infectious disease. We undertook analyses, using the best available data, to relate outbreaks of polio to the continued armed conflict in Afghanistan.

### Polio prevention efforts in Afghanistan

The Global Polio Eradication Initiative (GPEI), a project of the World Health Organization, coordinates the efforts of the Afghani government, UNICEF, and various NGOs to eradicate polio. The GPEI national team is responsible for policy, planning, and vaccine supply, while provincial teams are responsible for implementation, supervision, and monitoring of program activities. Through this network, over 2,700 vaccinators at 1,251 vaccination sites provide routine services to approximately 80% of the Afghani population [13].

In addition to routine services, the GPEI also engages in supplemental immunization activities including national and subnational immunization days. Organizing “days of tranquility” is a major part of the Afghani immunization campaign; these campaigns, which absorb time, political capital, and money, deliver a dose of oral polio vaccine to all children under 60 months of age, regardless of personal vaccination history. The GPEI also conducts “mop-ups:” children in the vicinity of a polio outbreak are revaccinated. National immunization days can involve almost 55,000 workers and volunteers [13]. The risk of violence has an impact on immunization activity and a better understanding of spatial patterns of violence (in this case IEDs) and disease can help inform future strategies in this or similar regions. Using maps to convey risk especially for policy and logistical decision making is common in the hazards and health literature. Understanding where and when polio is most likely to present itself in violence-afflicted regions could help target these immunization campaigns better.

## Methods

To explore the relationship between violent conflict and incident cases of polio in Afghanistan, we examined publicly available data collected in Afghanistan from 2004 to 2009 and conducted analyses to examine the geographical relationship between occurrence of violence and incidence of polio.

In Afghanistan, polio incidence is tracked by the World Health Organization surveillance programs. Acute flaccid paralysis surveillance (for all cases including but not limited to those caused by poliovirus) is conducted by a nationwide network of over 10,000 volunteers who report into a WHO surveillance system. These volunteers (which include allopathic practitioners, traditional healers, shrine keepers, and mullahs) report any cases of acute flaccid paralysis to one of 483 “focal points” (usually pediatricians), who are then responsible for investigation and sample collection. In over 80% of cases, samples are tested in provincial laboratories to identify wild-type poliovirus, vaccine-derived poliovirus, and related non-polio enteroviruses [13]. The WHO assesses the completeness of its surveillance program by monitoring the rate of non-polio acute flaccid paralysis. In a normal population, 1–2 cases per 100,000 children are expected. All areas, including those with the highest degree of violence, have consistently reached this benchmark each year, leading the Afghanistan Polio Eradication Initiative to conclude that the “surveillance network is functional even in [the worst security affected] districts.” [13] Since this benchmark was met, WHO officials infer that underreporting of polio cases is minimal. In addition, over-reporting is unlikely because most cases of acute flaccid paralysis undergo laboratory testing to confirm or reject the presence of polio [13].

To provide a spatial measure of violence, we use data from detonations of improvised explosive devices (IEDs). We chose IEDs as our geographic measure of violence because of the very precise spatial nature of these data and because the vast majority of coalition deaths have occurred from IEDs. A Wikileaks release in 25 July 2010 includes latitude and longitude for every IED detonation in Afghanistan from 2004–2009.The data displayed here represent the 3,414 detonations that occurred in 2009, the most recent year available. Although NATO and other military forces have declined to confirm or deny the accuracy of this data, The New York Times, The Guardian, and Der Spiegel have all concluded that it appears to be genuine [15].

To approximate the spatial distribution of polio cases, we relied on the only data available: reports from the GPEI in Afghanistan, which identified 13 “high-risk” districts that contained the majority of the polio cases in the country in 2010; these polio high-risk districts have remained stable from year to year during the period under consideration. Maps were generated using ARCGIS software (version 10, Esri: Redlands, CA). Districts were defined as “high-risk” based upon epidemiological data from 2010 that demonstrated the majority of confirmed cases originated from these areas [16].

To assess the quantitative spatial relationship between IED detonations and polio risk, we compare the mean number of IED detonations in polio high-risk districts as compared to non polio high-risk districts. We used the Wikileaks data to identify how many IED detonations occurred in each district each year, then we calculated the mean number of IED detonations per district. The difference in means between the mean number of IED detonations in polio high-risk districts and the mean number among the non polio high-risk districts was evaluated using a T-test.

## Results

**Fig 2:**
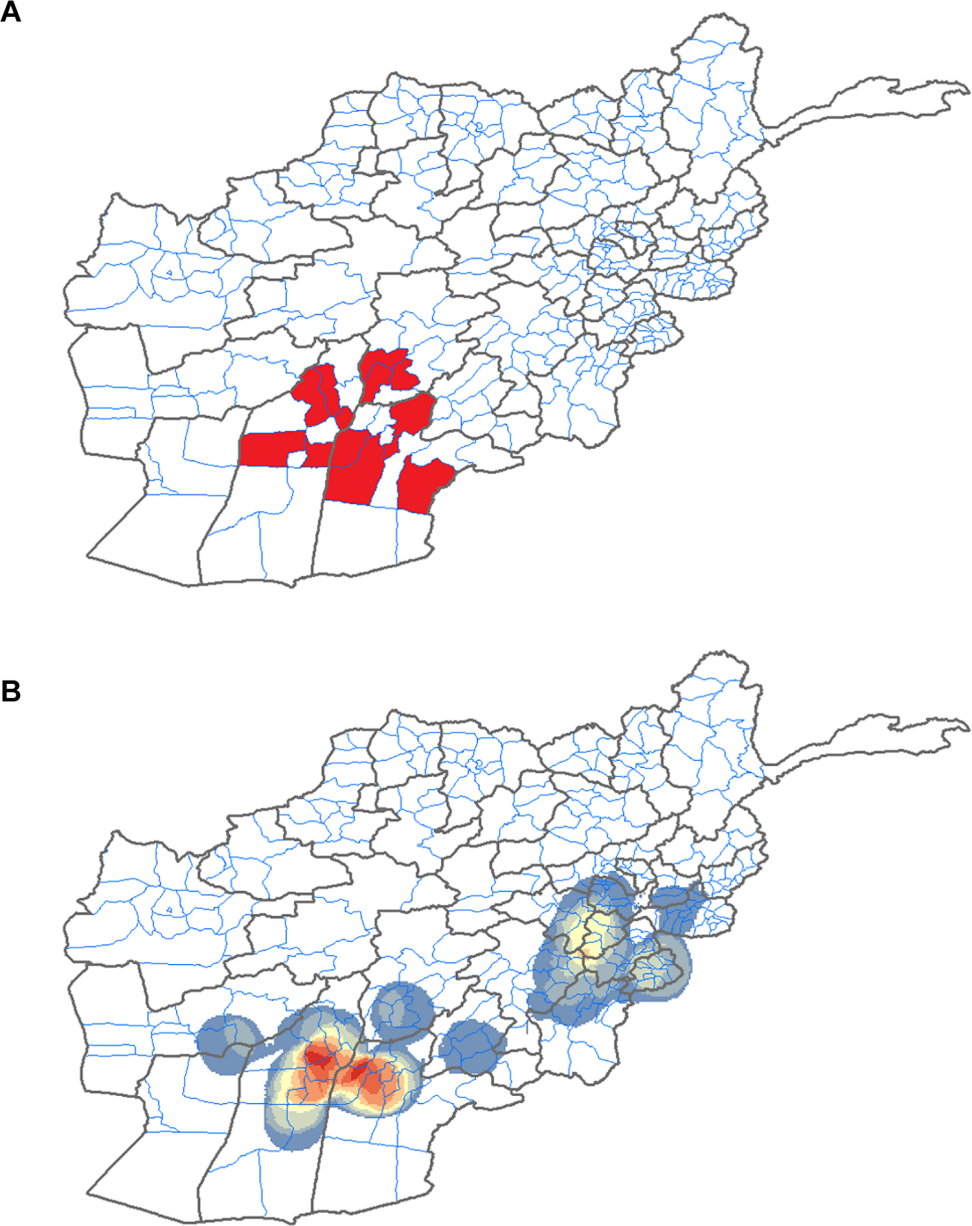
Greater IED density overlaps with high risk polio districts. (A) The thirteen “high-risk” districts, as identified by the Afghanistan members of the Global Polio Eradication Initiative. Adapted from GPEI 2010 Annual Report. (B) Incidence density map created by plotting 3,414 IED detonations from 2009.

Polio incidence is spatially associated with violence. The majority of polio cases in Afghanistan are found in a very small geographic area. Thirteen districts are responsible for 80–90% of polio cases in the entire country (Fig 2A), despite containing only 9% of the population [13]. Plotting the incidence density of IED detonations reveals the regions where violence is most concentrated (Fig 2B). Qualitatively, concentrated violence overlaps with high-risk for polio.

We demonstrate quantitatively that for each year 2004 through 2009, the districts identified as high-risk for polio had statistically significantly higher mean number of IED detonations per districts than the other districts of Afghanistan (p-values for t-tests range from 0.040 to 0.001, Table 1).

**Table 1:** Comparison of mean number of IED detonations in non-polio high-risk districts vs. polio high-risk districts, by year.

	Non-polio high-risk district (n = 315 districts)			Polio high-risk districts (n = 13 districts)			
Year	Total IEDs across districts	Mean IEDs/ district	(SD)	Total IEDs across districts	Mean IEDs/ district	(SD)	P-value for t-test
2004	160	0.51	(1.22)	34	2.62	(3.23)	0.0360
2005	233	0.74	(1.66)	61	4.69	(4.61)	0.0098
2006	562	1.78	(3.65)	150	11.54	(13.42)	0.0228
2007	907	2.88	(6.91)	251	19.31	(25.68)	0.0404
2008	1170	3.71	(9.64)	504	38.77	(37.26)	0.0055
2009	2525	8.02	(21.01)	1044	80.31	(60.13)	0.0010

## Discussion

We illuminate a substantial spatial relationship between violence and incident cases of polio in Afghanistan between 2004 and 2009. Identifying this relationship is an important first step in exploring the effects of armed conflict on the spread of polio. Our analysis, based on publicly available data, begins to describe the mechanism that links violence to incident polio, permitting public health practitioners to call on armed actors to change their behavior. We believe the most likely explanation for the relationship is that violence leads to reduced rates of polio vaccination, which is in turn responsible for increased polio incidence. This explanation is consistent with the apparent inverse relationship between violent incidents and regional vaccine coverage. Supporting this theory, a separate study from Pakistan and Afghanistan suggests that the increased numbers of polio cases in between 2001 and 2011 are unlikely to be a result of decreased vaccine efficacy, and instead the consequence of decreases in vaccination coverage [17]. Some spatio-temporal estimates of vaccine coverage exist, but this data is only specific to the regional level. Without being able to compare rates of immunity at the level of districts, these figures may mask stark disparities. [17]

Two factors contribute substantially to continued rates of low vaccination in conflict areas. First, vaccination campaign staff are being directly targeted by armed groups. The Annual Report on Polio Eradication from the Afghanistan Ministry of Public Health outlined several security incidents from 2010: the murders of the public health director of Kunduz province, direct threats to attack UN compounds in Gardez and Kandahar, and staff abductions in Khost and Paktya [13]. The Afghani government and international agencies have been forced to suspend operations or delay subnational immunization days in some regions [13]. On the other hand, other reports indicate that insurgent forces have cooperated with vaccination staff. In 2007, the International Committee of the Red Cross convinced Mullah Omar, the spiritual leader of the Taliban, to sign a letter to be carried by all vaccination staff, guaranteeing them safe passage through Taliban-controlled regions. However, Omar did not represent all anti-government elements; other groups must also be addressed through direct negotiation [18].

A second reason for low vaccination rates in conflict areas is that the conflict creates a lack of public trust in the governmental and international organizations that run vaccination campaigns. Without trust, a successful vaccination program is impossible. Indeed in some countries the polio vaccine program has become entwined in the political dynamics of an armed conflict, causing widespread distrust of the program in some communities. In Nigeria, anti-government Muslim groups viewed the polio program as a tool of Western imperialism [6]. In Pakistan, the CIA’s use of fake hepatitis vaccination programs in 2011 to gather intelligence raised suspicion of the polio program, and may have “contributed to subsequent decreasing vaccine confidence and polio-vaccine uptake in Taliban-sympathetic regions of both Pakistan and Afghanistan, even 1 year later” ([19], p461).

Our study, an ecologic examination of violence and polio, relies on country-level data, presented in aggregate based on annual totals. Due to the independent seasonal nature of both polio incidence and violence in Afghanistan, annual totals (as opposed to monthly or quarterly totals) are the most appropriate measure for our analysis. Further, an ecologic study was the best study design available for several reasons. On a purely pragmatic level, ecologic level data were the only data publically available. Additionally, IEDs and other means of violence tend to impact communities in addition to individuals, so assessing the level of violence in a country or province may be more relevant than any individual’s level of exposure to violence. Violence can damage infrastructure, cause suspension of vaccination activities, and influence the behavior of whole communities. Trying to understand or quantify the impact of a single death in such a context may not be as effective as examining aggregate totals of violence. We also posit that these preliminary findings should be used to leverage resources and data for more rigorous individual level studies, using a cohort or case-control study design.

Although we demonstrate that a greater mean number of IED detonations occurred in polio high-risk districts, we cannot perform a more detailed spatial analysis without additional polio case data. Also, to be counted in our analysis, the infection must have been detected by the WHO surveillance programs. While the WHO reports that their surveillance system is intact, if political insecurity and violence resulted in a breakdown of disease surveillance systems, our figures will be a conservative measure of polio. The true association between violence and polio would be even stronger if cases of polio went undetected as a result of violence.

Although we demonstrate a substantial spatial relationship between violence and incident polio cases, we cannot establish causality. In addition, other contributing factors may include increased disease exposure due to damaged water and sewage infrastructure. Our geographic analysis depends on the GPEI definition of high-risk polio districts; we lack data to control for differences between high-risk districts and non high-risk districts (such as likely vaccination coverage independent of armed conflicts).

We argue that the most likely driver for the relationship between violence and polio disease is disruption of vaccine coverage. Here we also consider a second possibility: vaccine efficacy may be subject to modulation by exposure to traumatic events. Little research investigates this effect directly, but several steps in the pathway have been examined. In 2010 in occupied Palestine, researchers found that children with high levels of exposure to traumatic events had constitutively elevated cortisol [20]. In separate research, elevated levels of stress hormones have been demonstrated to impact the peripheral T-cell profile of soldiers with post-traumatic stress disorder [21]. This possibility merits further investigation, because almost half of children with paralytic polio in Afghanistan have, by parents’ self-report, received four or more doses of the oral vaccine [11].

Our analysis brings new light to the public health situation of Afghanistan and other contexts of armed conflict. Polio outbreaks have occurred in Somalia in 2011 approximately two years after conflict escalated in 2009 with the new government and again in 2013, two years after Kenyan military forces entered Somalia in 2011 [22]. Likewise, the Syrian Civil War started in early 2011, and two years later there were 17 cases of polio [23]. In order to clarify the relationship between violence and polio, more precise measures of both violence and disease are needed. Now that we have demonstrated a quantitative geographic relationship between polio and violence, a more granular examination within districts is possible. In addition, qualitative research is essential to elucidate the mechanisms by which violence is associated with polio. Given the geographic relationship between violence and polio that we have demonstrated, the implications of these results for armed actors are two fold. First, they should support polio vaccine distribution efforts in communities exposed to violence. Second, they should take all available measures to avoid entangling the polio campaign in political dynamics of the armed conflict. Of note, NATO did develop a formal policy of “passive support” for the polio campaign in Afghanistan whereby NATO forces avoid all visible association with the campaign but at the same time take measures to support vaccine distribution such as participating in “days of tranquility.” This policy can serve as a model for other armed groups in polio-affected countries.
